# Neonatal Mortality and Associated Factors in the Neonatal Intensive Care Unit of Gadarif Hospital, Eastern Sudan

**DOI:** 10.3390/children9111725

**Published:** 2022-11-10

**Authors:** Mohammed Ahmed A. Ahmed, Hyder M. Mahgoub, Abdullah Al-Nafeesah, Osama Al-Wutayd, Ishag Adam

**Affiliations:** 1 Faculty of Medicine, Gadarif University, Gadarif 32211, Sudan; 2 Department of Pediatrics, Unaizah College of Medicine and Medical Sciences, Qassim University, Unaizah 56219, Saudi Arabia; 3 Department of Family and Community Medicine, Unaizah College of Medicine and Medical Sciences, Qassim University, Unaizah 56219, Saudi Arabia; 4 Department of Obstetrics and Gynecology, Unaizah College of Medicine and Medical Sciences, Qassim University, Unaizah 56219, Saudi Arabia

**Keywords:** NICU, birth asphyxia, preterm, neonatal mortality, Sudan

## Abstract

Background: Neonatal mortality is a serious public-health issue, especially in Sub-Saharan African countries. There are limited studies on neonatal mortality in Sudan; particularly, there are none on eastern Sudan. Therefore, this study aimed to determine the incidence, causes and associated factors for mortality among neonates admitted to the neonatal intensive care unit (NICU) of Gadarif Hospital, eastern Sudan. Methods: This retrospective study included 543 neonates admitted to the NICU of Gadarif Hospital, eastern Sudan, between January and August 2019. Data were obtained from the hospital record using a questionnaire composed of sociodemographic data, neonatal and maternal information and neonatal outcomes. Logistic regression analyses were performed and the adjusted odds ratio (AOR) and 95% confidence interval (CI) were calculated. Results: Of the 543 neonates, 50.8% were female, 46.4% were low birth weight (LBW), 43.5% were preterm babies and 27% were newborns admitted after caesarean delivery. The neonatal mortality before discharge was 21.9% (119/543) of live-born babies at the hospital. Preterm birth and its complications (48.7%), respiratory distress syndrome (33.6%), birth asphyxia (21.0%) and infection (9.0%) were the most common causes of neonatal mortality. In multivariable logistic regression analysis, preterm birth (AOR 2.10, 95% CI 1.17–3.74), LBW (AOR 2.47, 95% CI 1.38–4.41), low 5 min APGAR score (AOR 2.59, 95% CI 1.35–4.99) and length of hospital stay <3 days (AOR 5.49, 95% CI 3.44–8.77) were associated with neonatal mortality. Conclusion: There is an increased burden of neonatal mortality in the NICU of Gadarif Hospital, eastern Sudan, predominantly among preterm and LBW babies.

## 1. Introduction

Although there has been a substantial decrease in neonatal mortality worldwide, the issue remains. In 2018, 2.5 million infant deaths in the neonatal period (age <28 days) were reported [1]. The majority of adverse neonatal outcomes occur in Sub-Saharan Africa, with the least progress made in reducing neonatal morbidity and mortality compared to other countries [1,2]. Most neonatal deaths occur during the first week of life (early neonatal death) and these deaths could be prevented by accessing the optimum/adequate maternal and newborn health care [3]. According to the ‘Third Sustainable Development Goal’, there is a need to end preventable newborn deaths, with all countries aiming to reduce the neonatal mortality rate to/or <12 per 1000 live births by 2030 [4].

Intrapartum asphyxia, premature births, sepsis and complications during pregnancy were the main causes of neonatal mortality [5]. There is a wide range and variability in the incidence of neonatal mortality (4.0–28.0%) in different African countries [5,6,7,8,9,10,11,12,13], including lower gestational age [14], low birth weight (LBW) [13,14,15], not attending antenatal care (ANC) [16], not initiating exclusive breastfeeding [16], higher maternal age [17,18] and positive maternal HIV status [17], associated with neonatal mortality.

Although there are several studies conducted on neonatal morbidity and mortality in the Sub-Saharan African countries [5,6,7,9,10,11,12,13], there are limited studies on neonatal mortality in Sudan [18]; particularly, there are none on eastern Sudan. Investigating the epidemiology (incidence, causes and associated factors) of neonatal morbidity and mortality is required to generate data that might be useful for evidence-based intervention. The current study was conducted to determine the incidence, causes and associated factors for mortality among neonates admitted to the neonatal intensive care unit (NICU) of Gadarif Hospital, eastern Sudan.

## 2. Materials and Methods

### 2.1. Study Design and Setting

A retrospective study was conducted at the NICU of Gadarif Hospital, eastern Sudan, between January and 31 August 2019. Gadarif city is situated at a mean altitude of 496 m above sea level and lies between latitudes 14 and 16 North and longitudes 33 and 36 East. It has a population of 1,727,401 residents. It is 400 km from the capital of Sudan (Khartoum) and it is located on the Ethiopian border.

All medical files/records of the neonates admitted to the unit during the study period were reviewed. The NICU of Gadarif Hospital offers special care to neonates at risk or who are ill. The unit is supervised by 3 pediatricians, 8 general practitioners and 13 nurses. In addition to other basic newborn neonatal services, the NICU has the following facilities: nine incubators, supplemental oxygen administration, nine radiant warmers, five phototherapy machines, umbilical transfusion, nasogastric tube insertion, intravenous infusion, urinary catheterisation, lumbar puncture, necessary laboratory investigation and continuous positive airway pressure ventilation.

Data were obtained from the hospital records using a questionnaire composed of sociodemographic data and neonatal and maternal information, which included birth weight, length of hospital stay, duration of symptoms and signs, maternal age, gender, mode of delivery (vaginal or caesarean), APGAR score and gestational age. It also contains primary admission diagnosis, outcome and cause of death.

The data include all neonates delivered at the hospital and admitted to the NICU. Exclusion criteria include neonates with incomplete files, who were delivered outside the hospital and who were presented only for a brief observation.

#### 2.1.1. Sample Size Determination and Sampling Procedure

The consecutive sampling technique was followed. The sample size of 543 neonates was calculated based on the incidence of neonatal mortality reported in Ethiopia (20.0%) [19]. We assumed a ratio of 1:4 (incidence of mortality of 20.0%) between neonates who died and neonates who survived. Moreover, we assumed that 56.0% of the neonates who died were LBW babies and 40.0% of the neonates who survived were LBW babies (as risk factor for death among the neonates). This sample size had 80% power with a precision of 5%, assuming that 10% of the files would have incomplete data

#### 2.1.2. Operational Definitions

Preterm birth was defined if the gestational age was <37 weeks. LBW was defined if the birth weight was <2.5 kg. Birth asphyxia was diagnosed if there was one of the following signs: newborn was not breathing or gasping, breaths were < 30 per minute, APGAR score was < 7, presence of neurologic sequelae, namely seizures, coma and hypotonia or the newborn had multiple organ involvement (kidney, lungs, liver, heart and intestines) [20].

### 2.2. Ethics

The study received ethical approval from the Research Board at the Faculty of Medicine, University of Gadarif, Sudan. The reference number is 2019 ref16. As the data were collected retrospectively, patient consent was not required by the Research Board at the Faculty of Medicine, University of Gadarif, Sudan. Patient data were reserved, confidential and used only for research purposes. This study was conducted in accordance with the Declaration of Helsinki.

### 2.3. Statistics

Data analysis was performed using SPSS software ver. 22.0 (SPSS Inc., Chicago, IL, USA). Quantitative indices were checked for normality using the Shapiro–Wilk test. Quantitative and categorical variables were compared between the two groups using the *t*-test, non-parametric Mann–Whitney U test and chi-square test as applicable. Univariate and multivariate logistic regression analysis was performed with neonatal mortality as an independent variable (age, gender and gestational age). Variables with a *p* value of <0.20 in the univariate analysis were shifted to the multivariate model. Variance inflation factor (<4) was used to assess the presence of collinearity. Odds ratios (ORs) and 95% confidence intervals (95% CIs) were computed for each variable and were adjusted using a backward likelihood ratio. A two-tailed *p*  < 0.05 was considered significant.

## 3. Results

Data of 543 (50.8% female) neonates were collected and included in this analysis. The median (interquartile range (IQR)) maternal age was 27.0 (23.0–30.0) years and the number of children was 3 (2–5); median (IQR) gestational age was 37.0 (34.0–38.0) weeks and 236 (43.5%) were preterm births; median (IQR) birth weight of the neonates was 2500 (1500–3000) gm and 252 (46.4%) were LBW babies; and median (IQR) hospital stay was 3.0 (2.0–4.0) days and 206 neonates (37.9%) stayed <3 days. Overall, 246 (63.7%) neonates were delivered vaginally, whereas 197 (36.3%) were delivered via caesarean section (Table 1).

**Figure 1:**
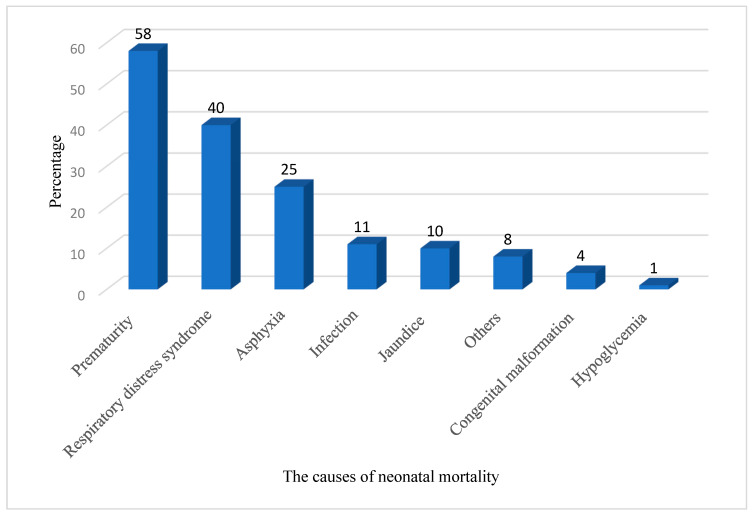
Common causes of neonatal mortality in the neonatal intensive care unit of Gadarif hospital.

Furthermore, 119 (21.9%) neonates died and the remaining survived from admission to discharge in the NICU of Gadarif Hospital. Preterm birth and its complications (48.7%), respiratory distress syndrome (33.6%), birth asphyxia (21.0%) and infection (9.0%) were the most common causes of neonatal mortality (Table 2, Figure 1). The reported congenital abnormalities were neural tube defect, congenital heart disease, Down syndrome and digestive tract obstruction.

### Factors Associated with Neonatal Mortality

**Figure 2:**
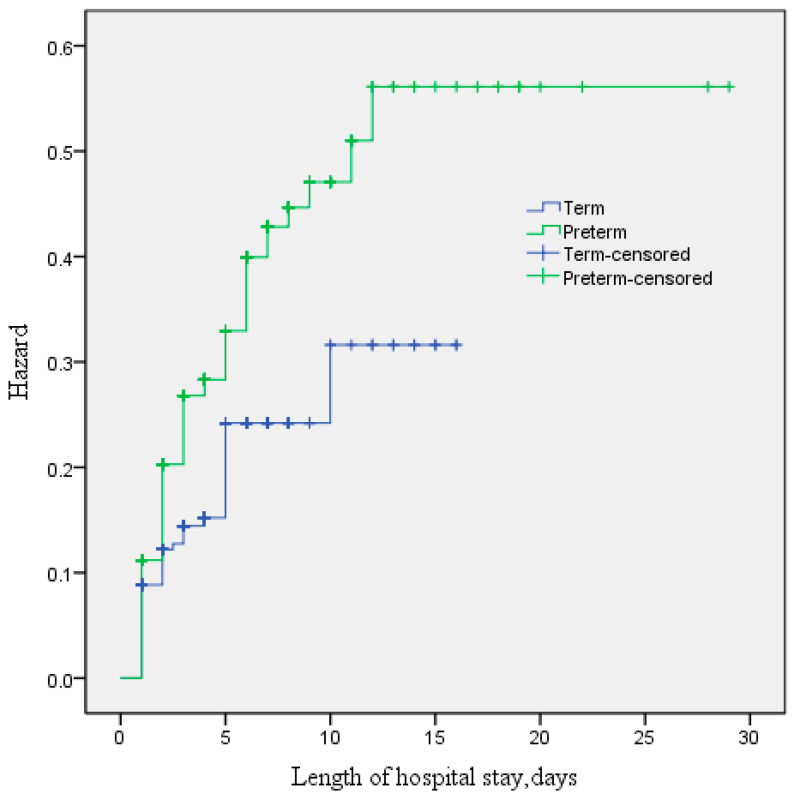
Kaplan–Meier curve to show the difference between preterm and full-term infants.

There was no collinearity between the covariates. In the multivariable logistic regression analysis, preterm birth (adjusted OR (AOR) 2.10, 95% CI 1.17–3.74), LBW (AOR 2.47, 95% CI 1.38–4.41), low 5 min APGAR score (AOR 2.59, 95% CI 1.35–4.99) and length of hospital stay <3 days (AOR 5.49, 95% CI 3.44–8.77) were associated with neonatal mortality (Table 3), Figure 2. However, maternal age, number of children, gender of the newborn and mode of delivery were not associated with neonatal mortality (Table 3).

## 4. Discussion

The main findings of the current study showed that the incidence of neonatal mortality in the NICU of Gadarif Hospital, eastern Sudan, was 21.9%, which was associated with preterm birth and low APGAR score. On the contrary, and surprisingly, a national survey in Sudan reported a low incidence (3.0%) of neonatal mortality [18]. The difference between our results and the results of the national survey in Sudan could be explained by the nature of the study, which was a hospital one (our study). Similar to our findings, Debre Markos, Ethiopia, reported a 21.3% incidence of neonatal mortality [16]. In addition, the incidence of neonatal mortality (21.9%) in our study is consistent with the findings of previous studies conducted in Ethiopia (20.0%) [5] and (23.1%) [6] and Nigeria and Kenya (18.7%) [14]. Different neonatal mortality rates were reported in different African countries, such as 5.7–23.3% in Ethiopia [9,10], 13–38% in Kenya [7,8], 15.7% in Cameroon [12], 18.8% in Nigeria [11] and 15% in Somalia [21]. On the contrary, the incidence of neonatal mortality in NICU in our study (21.9%) was much higher than that reported in Eritrea (6.5–8.2%) and [13,15] Burkina Faso (4.6%).

We observed that preterm birth and its complications, birth asphyxia and infection were the most common causes of neonatal mortality, which is in line with the study conducted in the SNCUs of Orotta National Maternity Referral Hospital, Eritrea [13], and the specialised NICU in Asmara, Eritrea [15]. In eastern Ethiopia [5] and Somalia [21], preterm birth, birth asphyxia and neonatal infection were the main causes of neonatal mortality and these factors were associated with intrapartum and early postpartum care. Hence, intrapartum and immediate newborn care practices should be optimized. According to the World Health Organization, almost half (47%) and three-quarters (75%) of all neonatal mortality occur in the first day and first week of life, respectively [22].

Our study indicated that preterm birth and LBW (2.10 and 2.47, respectively), as well as low APGAR score, were associated with increased risk of neonatal mortality, consistent with the findings of several studies conducted in many Sub-Saharan African countries, such as Eritrea [13,15], Ethiopia [5,6,16] and Nigeria [22].

These observations highlighted the importance of detecting the potential determining factors (mainly obstetrical) behind the pathogenesis of LBW and preterm birth, including optimizing the management of these factors. Moreover, preterm and LBW babies were susceptible to complications, such as asphyxia hypothermia and sepsis. Thus, proper neonatal care, including appropriate feeding, temperature maintenance and hygienic cord-cutting and covering, can substantially avert some of these effects and reduce mortality [22].

In the current study, newborns hospitalized for <3 days were at a 5.49-times higher risk of dying. In neighboring Ethiopia, newborns hospitalized for <3 days were 3.6-times more likely to die compared to those hospitalized for 4–7 days [22].

Moreover, in Eritrea, newborns hospitalized for >1 day in the NICU were at a lower risk of neonatal mortality [15]. In the Somali region of Ethiopia, newborns hospitalized for <2 days in the NICU were less likely to die (AOR 0.418) [22]. In the current study, there was no association between maternal age, gender of the newborn and mode of delivery and neonatal mortality. This is in line with the previous finding in Eritrea, in which none of the maternal conditions were associated with neonatal mortality [15]. On the contrary, a Sudan household survey analysis indicated that maternal age, male child and caesarean delivery were associated with neonatal mortality [22]. It is worth mentioning that several factors, such as feeding status [5], hypothermia [13], antenatal care [15], mother’s education and HIV status [17], which were not assessed in our study, were found to be predictors of neonatal mortality and if these were assessed, the results may have been different. Moreover, the difference in the sociographic characteristics, as well as other factors, such as HIV, has to be considered when comparing the findings of our study and those of others.

Nonetheless, this study has some limitations. First is the retrospective nature of the study and many variables were not assessed. Second, this study was hospital based; therefore, it might not reflect the exact situation at the community level and the outcomes of the neonates who were delivered were unknown.

## Figures and Tables

**Table 1:** Characteristics of newborns admitted to the NICU of Gadarif Hospital (N = 543).

Variables		Median	IQR
Maternal age, years		27.0	23.0–30.0
Number of children		3	2–5
Gestational age, weeks		37.0	34.0–38.0
Birth weight, kg		2.5	1.5–3.0
Duration of admission, days		3.0	2.0–4.0
		Frequency	Proportion
Mode of delivery	Vaginal	346	63.7
	Caesarean	197	36.3
Preterm birth	Yes	236	43.5
	No	307	56.5
LBW	Yes	252	46.4
	No	291	53.6
Low APGAR score	Yes	428	78.8
	No	115	21.2
Gender	Female	276	50.8
	Male	267	49.2
Length of hospital stay <3 days	Yes	206	37.9
	No	337	62.1

NICU, neonatal intensive care unit; IQR, interquartile range; LBW, low birth weight.

**Table 2:** Comparison between newborns who died and survived in the neonatal intensive care unit of Gadarif hospital (N = 543).

Variables		Survived	Died	*p*
Median (interquartile range)				
Maternal age, years		27.0 (7.0)	26.0 (8.0)	0.300
Number of children		3 (3)	3 (4)	0.421
Gestational age, weeks		37.0 (3.0)	35.3 (7)	<0.001
Birth weight, kg		2.6 (1.3)	1.8 (1.7)	<0.001
Duration of admission, days		4.0 (4.0)	2.0 (2.0)	<0.001
Frequency (proportion)				
Mode of delivery	Vaginal	260 (61.3)	86 (72.3)	0.031
	Caesarean	164 (38.7)	23 (27.7)	
Preterm birth	Yes	165 (38.9)	71 (59.7)	<0.001
	No	259 (61.1)	48 (40.3)	
low birth weight	Yes	175 (41.3)	77 (64.7)	<0.001
	No	249 (58.7)	42 (35.3)	
Low APGAR score	Yes	324 (76.4)	105 (88.2)	0.005
	No	100 (23.6)	14 (11.8)	
Gender	Female	221 (52.1)	55 (46.2)	0.255
	Male	203 (47.9)	64 (53.8)	
Length of hospital stay <3 days	Yes	129(30.4)	77(64.7	<0.001
	No	295(69.6)	42(35.3)	

**Table 3:** Logistic regression of factors associated with neonatal mortality in the neonatal intensive care unit of Gadarif hospital.

	Adjusted	
Variables	OR (95.5 CI)	*p*
Caesarean delivery	1.50 (0.92–2.46)	0.101
Preterm birth	2.10 (1.17–3.74)	0.012
low birth weight	2.47 (1.38–4.41)	0.002
Low APGAR score	2.59(1.35–4.99)	0.004
Length of hospital stay <3 days	5.49 (3.44–8.77)	<0.001

OR, odds ratio; CI, confidence interval.

## Data Availability

The datasets used and/or analyzed during the current study are available from the corresponding author on reasonable request.

## Abbreviations

ANC, Antenatal Care; AOR, Adjusted Odds Ratio; MDG, Millennium Developmental Goal; NICU, Neonatal Intensive Care Unit; NMR, Neonatal Mortality Rate; RDS, Respiratory Distress Syndrome; WHO, World Health Organization.
